# Diverse Anti-Tumor Immune Potential Driven by Individual IFNα Subtypes

**DOI:** 10.3389/fimmu.2020.00542

**Published:** 2020-04-03

**Authors:** Anthony C. Buzzai, Teagan Wagner, Katherine M. Audsley, Hannah V. Newnes, Lucy W. Barrett, Samantha Barnes, Ben C. Wylie, Shane Stone, Alison McDonnell, Vanessa S. Fear, Bree Foley, Jason Waithman

**Affiliations:** ^1^Telethon Kids Cancer Centre, Telethon Kids Institute, The University of Western Australia, Nedlands, WA, Australia; ^2^PYC Therapeutics, QEII Medical Centre, Harry Perkins Institute for Medical Research, Nedlands, WA, Australia; ^3^National Centre for Asbestos Related Diseases, QEII Medical Centre, The University of Western Australia, Nedlands, WA, Australia

**Keywords:** interferon subtypes, adoptive cell therapy, tumor microenvironment, CD8^+^ T cells, immunotherapy

## Abstract

Immunotherapies harnessing T cell immunity have shown remarkable clinical success for the management of cancer. However, only a proportion of patients benefit from these treatments. The presence of type I interferon (IFN) within the tumor microenvironment is critical for driving effective tumor-specific T cell immunity. Individuals can produce 12 distinct subtypes of IFNα, which all signal through a common receptor. Despite reported differences in anti-viral potencies, the concept that distinct IFNα subtypes can improve anti-cancer treatments remains unclear. We tested whether expression of unique IFNα subtypes confined to the tumor microenvironment enhances tumor control. This was systematically evaluated by transplantation of B16 murine melanoma cells secreting five unique IFNα subtypes (B16_IFNα2; B16_IFNα4; B16_IFNα5; B16_IFNα6; B16_IFNα9) into a pre-clinical murine model. We show that IFNα2 and IFNα9 are the only subtypes capable of completely controlling tumor outgrowth, with this protection dependent on the presence of an adaptive immune response. We next determined whether these differences extended to other model systems and found that the adoptive transfer of tumor-specific CD8^+^ T cells engineered to secrete IFNα9 delays tumor growth significantly and improves survival, whereas no enhanced survival was observed using T cells secreting IFNα4. Overall, our data shows that the expression of distinct IFNα subtypes within the tumor microenvironment results in different anti-tumor activities, and differentially affects the efficacy of a cancer therapy targeting established disease.

## Introduction

Cancer immunotherapy strategies have proven successful in the treatment of several advanced malignancies. However, despite astonishing efficacy in some patients with metastatic melanoma, <20% of patients experience durable responses (1–3). One reason behind the lack of treatment efficacy is the immunosuppressive tumor microenvironment encountered, inhibiting the ability of host immunity to eliminate malignant cells (4). Thus, the development of new and improved strategies to mitigate tumor immunosuppression and enhance anti-tumor immunity to solid cancers are warranted.

It has been shown that the presence of type I interferon (IFN) within the tumor microenvironment favors effective immune responses (5, 6). The mechanisms by which type I IFNs mediate these effects are complex and not completely understood. Initially, type I IFNs were reported to act solely on tumor cells to abrogate cellular proliferation (7). However, it is now clear that the anti-tumor activity of type I IFNs relies heavily on their capacity to modulate immunity (8). Furthermore, endogenous type I IFN signaling is indispensable for the therapeutic efficacy of many anti-cancer treatments such as radiotherapy (6), chemotherapy (9), and modern immunotherapies (10). Type I IFNs have the capacity to modulate immunity in a variety of different ways (11). For example, they have been shown to enhance local CD8^+^ T cell immunity by recruiting cross-presenting CD8α^+^ DCs to tumors (12). In addition, selective loss of type I IFN signaling on cross-presenting DCs results in the progression of highly immunogenic tumors, suggesting that type I IFNs are critical for efficient tumor surveillance (13). It has also been demonstrated that type I IFNs augment the activity of NK cells (14) and B cells (15), providing a comprehensive immune response against cancer. Thus, the presence of type I IFNs within the local tumor microenvironment is integral to tumor control (5).

While previous studies have highlighted the importance of type I IFN in mediating effective anti-tumor responses, these studies primarily focus on the type I IFN family as a whole (5, 6, 8, 12, 13). However, the human genome encodes 16 type I IFNs that includes 12 unique functional IFNα subtypes. Although it remains unclear why numerous IFNα subtypes have been conserved throughout evolution, the manner in which some IFNα subtypes have evolved under strong purifying selection indicates that their functions are not all redundant (16). Differential anti-viral activities of the IFNα subtypes have been reported both directly on infected cells, and indirectly by modulating the immune response against a variety of viral infections (17–19). In addition, type I IFN subtypes can differentially activate members of the MAPK and STAT pathways resulting in different apoptotic and anti-proliferative effects in erythroleukaemia cells (20). However, there is a paucity of information regarding the role of different IFNα subtypes in mediating the immune response against solid tumors.

To date only the IFNα2 subtype has been used routinely in the clinic, resulting in increased relapse-free survival rates across a range of cancers (21) including metastatic melanoma (22). However, there was no change in overall survival. The possibility exists that many of the remaining untested IFNα subtypes may drive more potent anti-cancer activities. To investigate this further, we determined whether forced expression of distinct IFNα subtypes within the tumor microenvironment promotes enhanced tumor control in a murine model of melanoma. Five individual IFNα subtypes were tested in this model and induced two divergent responses. Whilst all IFNα subtypes restricted tumor growth over time, only local secretion of IFNα2 and IFNα9 could completely control the outgrowth of B16 tumors. This intriguing result provides evidence that the IFNα subtypes cluster into different biologically active anti-cancer classes. This concept is further supported in another model system of standard anti-cancer therapy, adoptive cell therapy (ACT), where T cells secreting IFNα9 are significantly superior against established tumors when compared to standard T cell therapy or T cells secreting IFNα4. Collectively, our findings provide a precedence for future strategic research to dissect the complex family of IFNα subtypes, and optimize the utilization of type I IFNs to improve cancer treatment protocols.

## Materials and Methods

### Cell Lines

B16-F10 (B16) murine melanoma cells were purchased from the ATCC and routinely passaged and cultured at 70–80% confluency in RPMI media (Life Technologies) supplemented with 10% FCS (Sigma-Aldrich), 2 mM L-glutamine, 50 μM 2-mercaptoethanol, 100 μg/mL streptomycin and 100 U/mL penicillin (all Life Technologies) (R10 media) at 37°C, 5% CO_2_. HEK293T cells and L929 cells were similarly passaged in DMEM media (Life Technologies) supplemented with 10% FCS, 100 μg/mL streptomycin and 100 U/mL penicillin.

### Plasmid Constructs and Transduction of B16 Cell Lines

The genes for murine IFNα2, IFNα4, IFNα5, IFNα6, and IFNα9 were amplified from the pkCMVint mammalian expression vector (18) and subcloned into the retroviral vector, pMIG, which also contained *IRES, GFP*, and *Amp*^*R*^ genes under a LTR promoter (Supplementary Figure 1A). Plasmid preparations were acquired from Terrific Broth cultures of transformed JM109 *E.Coli* (Promega) using standard DNA purification procedures with Lithium Chloride precipitation. B16 cells were transduced as described previously (23). Briefly, retroviruses were generated by transfecting the 293T cell line with pMIG-IFNα, pMD.old.gag.pol, and pCAG-VSVG. B16 cells were next transduced with 1 mL filtered retroviral supernatant in the presence of 8 μg/mL polybrene (Sigma-Aldrich). Transduced GFP^+^ cells were sorted by FACS to establish purified GFP^+^ cell lines. Transduced GFP^+^ cells were sorted using a BD FACSAria III cell sorter (BD Biosciences) to select stable B16_IFNα cell lines.

### IFNα Bioassay

Bioactive IFNα was confirmed using an *in vitro* IFN bioassay (24). Supernatants harvested from transduced B16 cell lines were treated at pH 2 for 1 h at −20°C to remove acid-labile proteins then neutralized to pH 7. Supernatants were centrifuged at 2,400 × g for 5 min followed by a further high-speed centrifugation at 22,000 × g for 15 min to remove cellular debris. Activity of IFNα was determined by exposing L929 cells to the acid-treated supernatants serially diluted across the plate. After 24 h, encephalomyocarditis virus (EMCV) was added to each well. Following a further 24 h incubation, end-point titres were defined as the dilution giving a 50% reduction in cytopathic effect (CPE) of the L929 cells. Bioactive IFNα titers were determined by comparing the CPE of the supernatants from each B16_IFNα cell line to the NIH IFNα/β standard (1,000 IU/mL).

### Co-culture Experiments

To assess whether the IFNα secreted by the B16_IFNα cells impeded the ability of bystander B16 cells to proliferate, B16_GFP or B16_IFNα cells were mixed at a 1:1 ratio with B16_Cherry bystander cells and labeled with violet proliferation dye (VPD) 450 (BD Biosciences) as per manufacturer's instructions before 5 × 10^4^ cells were seeded in one well of a 6-well-plate. Five days later, mixed B16 cell cultures labeled with proliferation dye were harvested and the level of VPD450 on both engineered B16_IFNα cells and bystander B16_Cherry cells was analyzed by flow cytometry using the BD LSRFortessa™. To assess the expression of MHC-I alleles, B16_GFP and B16_IFNα cells were seeded at a 1:1 ratio with B16_Cherry bystander cells in one well of a 6-well-plate. Seven days later, 5 × 10^4^ co-cultured B16 cells were seeded in one well of a 6-well-plate and left to adhere overnight. The next day, the media was replaced with R10 media supplemented with 10 ng/mL IFNγ (Shenandoah). After 48 h of IFNγ stimulation, the cells were harvested and stained with anti-mouse H-2D^b^ (KH95, 1:100) and anti-mouse H-2K^b^ (5F1, 1:200) and analyzed by flow cytometry.

### Mice

C57BL/6 mice were purchased from the Animal Resources Center, Western Australia. Type I IFN receptor knockout mice (IFNAR1^o/o^) (25), Recombination Activating Gene knockout mice (RAG1^o/o^) (26) and gBT.I mice (27) were bred at the Telethon Kids Institute. Animals were housed under pathogen-free conditions and all studies were approved by the Institute's Animal Ethics Committee (AEC) (AEC#252, AEC#289, and AEC#325).

### Tumor Challenge

Mice were injected subcutaneously with 5 × 10^5^ cells in 50 μL of RPMI media. For the mixed cell line experiment, 4.5 × 10^5^ B16_IFNα cells were mixed with 5 × 10^4^ B16_Cherry cells. Tumor size was monitored using calipers and tumor volume was calculated using the following formula: (length (mm) × width (mm)^2^)/2. Mice with tumors >1,000 mm^3^ were euthanised. Tumor-free mice were defined as mice with no palpable masses.

### Adoptive Transfer of gBT.I Cells Secreting IFNα

gBT.I cells were activated for 24 h in R10 media supplemented with 0.5 μg/mL anti-CD3 (BD Biosciences), 0.5 μg/mL anti-CD28 (BD Biosciences), 100 U/mL IL-2 (PrepoTech), and 2 ng/mL IL-7 (PrepoTech). The following day, cells were purified by a Lymphoprep™ density gradient and then transduced with previously generated retroviral supernatant using spinfection for 1 h at 2,000 × g in RetroNectin® (Takara Bio) coated plates. This spinfection was repeated the next day. Following transduction, gBT.I cells were expanded in R10 media with 100 U/mL IL-2 and 2 ng/mL IL-7 for 5 days. Following expansion, 3 × 10^6^ transduced gBT.I cells were intravenously transferred into irradiated (500 rads) recipients that were challenged 4 days prior with 5 × 10^5^ B16_gB cells.

### Statistical Analysis

All statistical analyses were performed using GraphPad (Graphpad Software Inc. v7.0a). Comparison of proliferation rate and MHC-I allele expression was assessed using a one-way ANOVA. Difference in tumor growth was compared using repeated-measure two-way ANOVA (mixed-model) followed by Bonferroni *post hoc* test. Differences in survival and tumor incidence was compared using the Log-Rank Mantel-Cox test. Statistical significance was indicated as ^*^p < 0.05, ^**^p < 0.01, ^***^p < 0.001, and ^****^p < 0.0001.

## Results

### Generation of B16 Cell Lines Secreting Functional IFNα Subtypes

**Figure 1:**
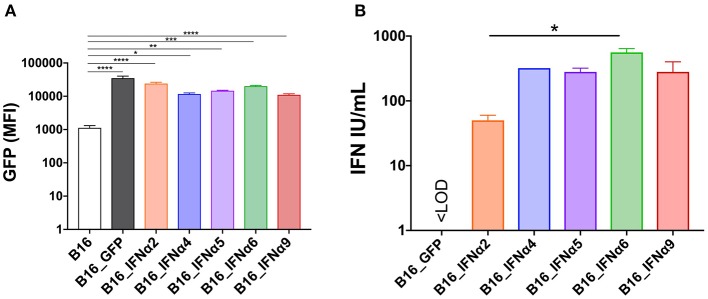
*In vitro* characterization of B16_IFNα cell lines. B16 melanoma cells were engineered to express the fluorescence reporter GFP and secrete IFNα. **(A)** Mean fluorescence intensities (MFI) of GFP between the engineered B16 cell lines (mean ± SEM). **(B)** IFN titer determined by a bioassay using supernatants derived from the engineered B16 cell lines (mean ± SEM). Data was pooled from two independent experiments and compared using one-way ANOVA, *p < 0.05, **p < 0.01, ***p < 0.001, and ****p < 0.0001.

To determine whether distinct IFNα subtypes differ in their capacity to modulate anti-tumor responses, we first engineered the B16 murine melanoma cell line to express discrete IFNα subtypes. Five different *IFNA* genes were selected and transduced into B16 cells using retroviruses generated with pMIG_IFNα vectors (herein: B16_IFNα2; B16_IFNα4; B16_IFNα5; B16_IFNα6; B16_IFNα9) and collectively referred to as B16_IFNα cells. A control cell line, B16_GFP, was also generated using the pMIG vector alone. The IFNα subtypes were selected based on unique characteristics that may correlate with potential differences in biological function including chromosomal location, direction of transcription, variations in amino acid sequence, and overall length of the secreted protein (Supplementary Figures 1B,C). We next conducted a series of experiments to determine if these recombinant cell lines were producing biologically active IFNα at similar doses. As IFNα expression was driven by the same promoter as GFP in the engineered cell lines, we measured GFP expression by flow cytometry. Across all the engineered B16_IFNα cell lines, GFP expression was comparable (Figure 1A). Currently, there is no single, absolute assay for measuring individual IFNα proteins. The most widely used method to determine IFNα biological activity and dose range is a cytopathic protective effects (CPE) assay. This assay detects the ability of titrated IFNα test samples to prevent viral infection against known dilutions of an international standard supplied by the NIH. To this end, acid-treated supernatants from recombinant B16 cell lines were titrated on L929 cells prior to infection with a single concentration of encephalomyocarditis virus (EMCV). As expected, supernatant from the B16_GFP cells did not protect L929 cells from EMCV-induced CPE, demonstrating that these cells were not producing detectable levels of IFNα (Figure 1B). In contrast, supernatant from B16_IFNα cell lines significantly protected L929 cells from EMCV-induced CPE (*p* < 0.0001), confirming secretion of bioactive IFNα. Dose quantification was determined against the titrated international standard, identifying cell lines within equivalent dose ranges, with the exception of B16_IFNα6 cells inducing a higher IFNα titer compared to the B16 cells secreting IFNα2 (*p* < 0.05). To determine if the constitutive production of IFNα by each of the B16_IFNα cells affected cell proliferation, B16_IFNα cell lines were co-cultured with wild type (WT) B16 melanoma cells expressing mCherry (Supplementary Figure 2). There was no significant differences detected in the proliferation rate of either the engineered B16_IFNα cells or the WT B16 cells. In addition, the secreted IFNα4, IFNα5, and IFNα9 could upregulate H-2K^b^ on both B16_IFNα and WT B16 melanoma cell lines (Supplementary Figure 3). Therefore, all B16_IFNα cell lines were secreting biologically active IFNα, which did not directly impede the proliferation of B16 tumor cells themselves and in some conditions upregulate the expression of H-2K^b^.

### IFNα Subtypes Have Different Anti-tumor Effects *in vivo*

**Figure 2:**
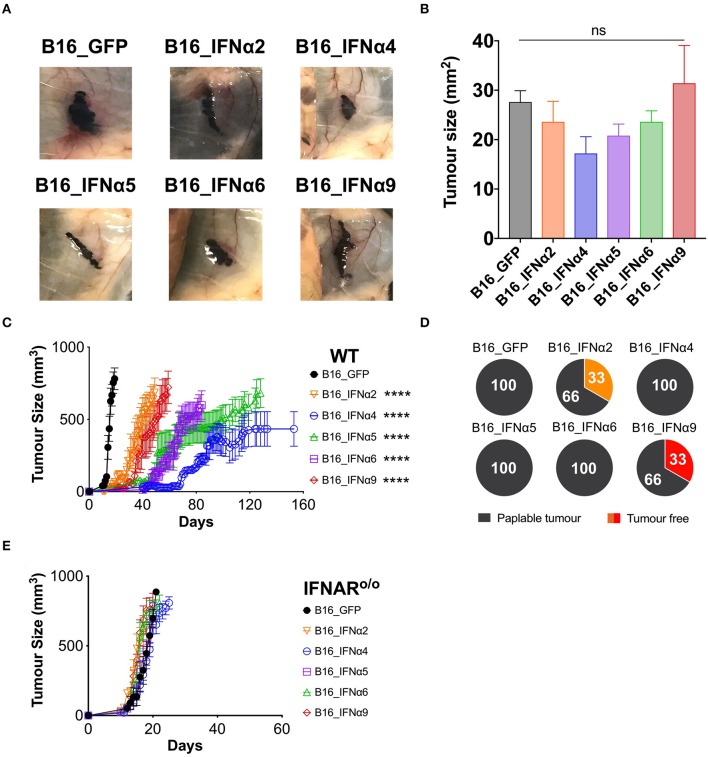
IFNα subtypes significantly delay tumor growth in WT mice. WT mice were inoculated subcutaneously with 5 × 10^5^ B16_GFP or B16_IFNα cells. **(A)** Representative images of subcutaneous tumors 8 days post-tumor inoculation (*n* = 5 per group). **(B)** Tumor area of B16 tumors (mean ± SEM) harvested 8 days post-tumor inoculation (*n* = 5 per group). **(C)** Tumor growth was measured over time. Each point signifies mean ± SEM combined from four independent experiments (*n* = 10–18 per group). **(D)** Proportions of WT mice that developed palpable tumors over time from four independent experiments (*n* = 10–18 per group). **(E)** IFNAR ^o/o^ mice were inoculated subcutaneously with 5 × 10^5^ B16_GFP or B16_IFNα cells. Tumor growth was measured over time. Each point signifies mean ± SEM from two independent experiments (*n* = 10–12 per group). Tumor growth curves of B16_GFP vs. each B16_IFNα were compared using repeated-measure two-way ANOVA (mixed-model) followed by the Bonferroni *post hoc* test, ****p < 0.0001.

Following the demonstration that the B16_IFNα cell lines produce functional IFNα at similar doses, we sought to determine if distinct IFNα subtypes differed in their capacity to mediate anti-tumor responses *in vivo*. C57BL/6 WT mice were challenged with either B16_GFP control cells or each of the individual B16_IFNα cell lines (Figure 2, Supplementary Figure 4). Eight days post-tumor inoculation, macroscopic tumors were present in all cohorts (Figure 2A) and no significant difference in tumor size between any of the groups was detected (Figure 2B). This data indicates that all inoculated cell lines have the capacity to establish and propagate *in vivo*. Mice inoculated subcutaneously with B16_GFP cells rapidly developed palpable solid tumor masses within 11 ± 1 days, and within 17 ± 2 days maximum tumor burden (Figure 2C), similar to growth kinetics observed following inoculation with the B16-F10 parental cell line (data not shown). In comparison, tumor development was significantly delayed in mice challenged with either B16_IFNα2 (22 ± 9 days, *p* < 0.0001), B16_IFNα4 (57 ± 13 days, *p* < 0.0001), B16_IFNα5 (48 ± 7 days, *p* < 0.0001), B16_IFNα6 (45 ± 12 days, *p* < 0.0001), or B16_IFNα9 cells (29 ± 8 days, *p* < 0.0001). Interestingly, B16 tumor growth was differentially controlled between the various IFNα subtypes. IFNα4, IFNα5, and IFNα6 restricted the progression of B16 tumors to a greater extent than IFNα2 or IFNα9. However, whilst the majority of mice challenged with B16 cells secreting IFNα2 and IFNα9 develop tumors at a faster rate than the other subtypes, 33% of mice in the IFNα2 and IFNα9 cohorts failed to develop palpable tumors (Figure 2D). This is in stark contrast to mice receiving B16_IFNα4, B16_IFNα5, or B16_IFNα6 cells, where tumor development was observed in 100% of the cohorts. Therefore, while all IFNα subtypes examined demonstrate anti-tumor activity, the IFNα subtypes have contrasting effects on melanoma formation and overall tumor progression.

IFNα subtypes can either exert their anti-tumor effects directly on tumor cells to inhibit proliferation and/or indirectly by acting through host cells to modulate anti-tumor immunity. To determine if IFNα exerts its effects solely on the tumor cells in our model, we assessed the capacity of B16_IFNα tumors to grow in IFNAR^o/o^ mice (25). These IFNAR^o/o^ mice lack the receptor through which all type I IFNs signal, thus any restriction of B16 tumor growth in these mice can only be attributed to the direct action of IFNα on the tumor cells from which they are secreted. IFNAR^o/o^ mice inoculated subcutaneously with B16_GFP or each of the B16_IFNα cell lines all rapidly developed palpable masses (Figure 2E) suggesting that the tumor cells themselves are not direct targets of IFNα.

### IFNα2 and IFNα9 Enhance Anti-tumor Immunity to Bystander WT Tumors

**Figure 3:**
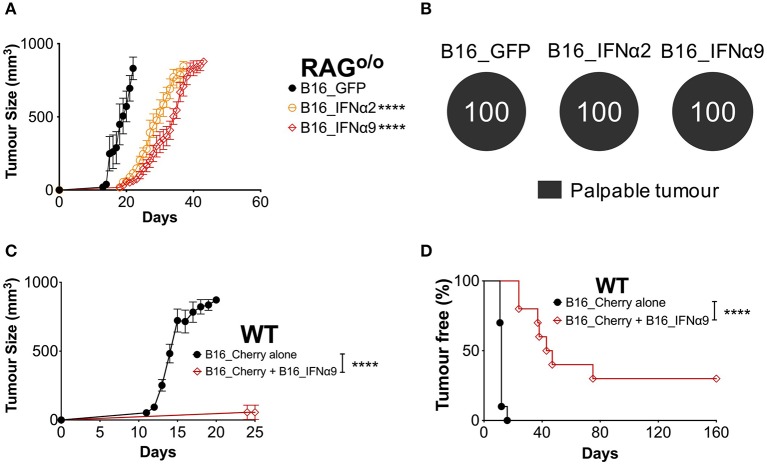
IFNα9 enhances anti-tumor immunity against bystander WT B16 tumors. **(A)** RAG^o/o^ mice were inoculated subcutaneously with 5 × 10^5^ B16_GFP or B16_IFNα cells. Tumor growth was measured over time. Each point signifies mean ± SEM from two independent experiments (*n* = 10–12 per group). Tumor growth curves of B16_GFP vs. each B16_IFNα were compared using repeated-measure two-way ANOVA (mixed-model) followed by the Bonferroni *post hoc* test, ****p < 0.0001. **(B)** Proportions of RAG^o/o^ mice that developed palpable tumors over time. **(C)** Tumor growth and **(D)** incidence of WT mice inoculated subcutaneously with 5 × 10^4^ bystander WT B16_Cherry cells alone or 5 × 10^4^ B16_Cherry cells mixed with 4.5 × 10^5^ B16_IFNα9 cells. Data combined from two independent experiments (*n* = 10 per group). B16_Cherry vs. B16_Cherry + B16_IFNα9 tumor growth curves were compared using repeated-measure two-way ANOVA (mixed-model) followed by the Bonferroni *post hoc* test and tumor incidence was compared using the Log-Rank Mantel-Cox test, ****p < 0.0001.

To determine whether the complete control observed in a proportion of WT mice inoculated with B16_IFNα2 and B16_IFNα9 cells required an intact immune system, we compared tumor burden in RAG^o/o^ mice and WT mice. RAG-deficient mice lack the recombinase machinery required to initiate V(D)J recombination that diversifies the T- and B cell repertoire. As such, RAG^o/o^ mice do not produce mature T and B cells and are incapable of mounting adaptive immune responses. Tumor growth was faster in RAG^o/o^ mice when compared to WT mice, however we still observed a delay in the growth of B16_IFNα tumors as compared to mice bearing B16_GFP tumors (Figure 3A, Supplementary Figure 5). However, in stark contrast to WT mice, where we observed a proportion of mice failing to develop a palpable tumor, all RAG^o/o^ mice that were challenged with either B16_IFNα2 or B16_IFNα9 developed palpable tumors (Figure 3B). This loss of protection against tumor growth in RAG^o/o^ mice suggests an important role for the modulation of the adaptive immune response by IFNα2 and IFNα9 within the local tumor microenvironment.

We next sought to determine if this immune-mediated protection by certain IFNα subtypes could be effective against bystander WT melanoma cells that do not secrete IFNα. WT mice were injected subcutaneously with either WT B16_Cherry cells or a mixture of B16_Cherry cells and B16_IFNα9 cells (Figures 3C,D). Within 16 ± 1 days, maximum tumor burden was reached in all WT mice challenged with B16_Cherry cells alone (Figure 3C). In contrast, tumor growth was significantly restricted (*P* < 0.0001) in WT mice injected with the mixture of B16_Cherry cells and B16_IFNα9 cells with maximum tumor size being reached in 49 ± 6 days. Additionally, whilst all WT mice implanted with B16_Cherry cells alone developed tumors within 12 days (Figure 3D), the majority of mice challenged with both B16_Cherry cells and B16_IFNα9 cells developed tumors between 35 and 80 days post-inoculation (41.1 ± 7 days, *p* < 0.0001). Remarkably, similar to what we had observed with B16_IFNα9 cells alone, 30% of these mice challenged with the mixed tumor cell populations remained tumor-free, highlighting the improved immunity afforded by IFNα9 is effective on bystander tumor cells.

### IFNα9, but Not IFNα4, Enhances ACT Efficacy Against Established Tumors

**Figure 4:**
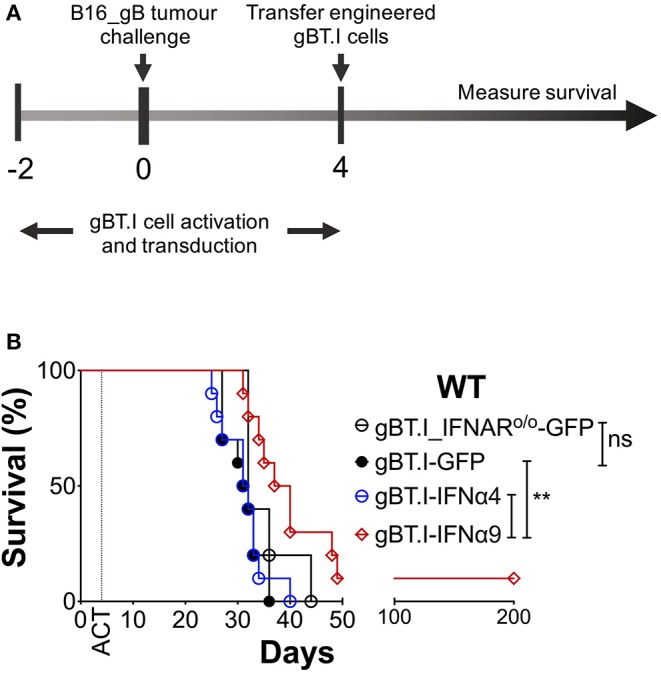
Delivery of IFNα9 into the tumor microenvironment by gB-specific CD8^+^ T cells improves survival. **(A)** gBT.I cell activation and transduction began 2 days prior to subcutaneous tumor challenge of WT mice with 5 × 10^5^ B16_gB cells. Four days post-tumor inoculation, mice were subjected to 500 rads total body irradiation before receiving 3 × 10^6^ gBT.I cells (gBT.I-GFP) or gBT.I cells lacking the IFNAR (gBT.I_IFNAR^o/o^-GFP) not secreting IFNα, or secreting IFNα4 or IFNα9. **(B)** Survival was monitored over time and data pooled from two independent repeats (*n* = 5–10 mice per group). The IFNα9 cohort was compared to GFP alone and IFNα4 cohorts using the Log-Rank Mantel-Cox test, **p < 0.01 for both comparisons.

To further demonstrate that the protection afforded by IFNα9 is immune-mediated and not intrinsic to the B16 cells expressing IFNα9, we used a model of ACT to deliver IFNα9 to the tumor microenvironment. In this model, TCR transgenic CD8^+^ T cells (gBT.I) (27), specific for HSV-derived glycoprotein B (gB), were engineered to either express GFP (vector control) and/or secrete IFNα9 or IFNα4. WT mice were challenged with B16 tumors expressing the model neoantigen gB (B16_gB), and 4 days later received a lymphodepleting dose of irradiation followed by adoptive transfer of the engineered gBT.I cells (Figure 4A). Treatment of WT mice bearing B16_gB tumors with effector gBT.I cells solely expressing GFP naturally offered a degree of therapeutic benefit, with all mice surviving for 31 ± 1 days (Figure 4B, Supplementary Figure 6). This was comparable to mice treated with gBT.I cells lacking the IFNAR (35 ± 2 days), demonstrating that endogenous IFN induced by irradiation was not contributing to the therapeutic efficacy afforded by the gBT.I cells. Similarly, mice infused with effector gBT.I cells secreting IFNα4 lived for 31 ± 1 days. In contrast, increased survival was observed in WT mice receiving effector gBT.I cells secreting IFNα9 (54 ± 16 days) as compared to mice treated with either non-IFNα-secreting (GFP, *p* = 0.0028) or IFNα4-secreting (*p* = 0.0059) gBT.I cells. Notably, one mouse treated with IFNα9 expressing gBT.I cells remained tumor-free for 200 days post-tumor inoculation. This data indicates that secretion of IFNα9 by T cells in the tumor microenvironment is beneficial to overall survival in tumor-bearing mice.

## Discussion

Using a systematic approach, we provide novel evidence that distinct IFNα subtypes have different immunomodulatory roles against a solid tumor. Of the IFNα subtypes tested in this study, we report a clear split between the subtypes anti-tumor activity, with IFNα4, IFNα5, and IFNα6 delaying tumor growth for over 100 days, whereas IFNα2 and IFNα9 are able to modulate the immune system to provide complete protection against tumor challenge in a proportion of mice. The adaptive immune system is critical for this protection, and this effect is not only intrinsic to B16 cells secreting IFNα as protection was also afforded to bystander B16 melanoma cells. Furthermore, we demonstrate in another model that local delivery of IFNα9, but not IFNα4, significantly enhances tumor control by bolstering the capacity of transferred tumor-specific T cells to target melanoma.

Since the discovery of IFNα over 60 years ago, this family of cytokines has attracted considerable attention for their anti-viral properties (28). More recently, the capacity of the IFNα family to regulate tumor growth has become an important focus of cancer treatments due to the important role they play during radiation therapy (6), chemotherapy (9), and immunotherapy (10). The vast majority of studies to date have not focussed on individual members of the IFNα family, despite the genome encoding for 12 distinct IFNα subtypes in humans (29, 30) and 14 subtypes in mice (30, 31). Variations in the amino acid sequences between the distinct IFNα subtypes affects their affinity to bind the IFNAR (29) resulting in differential downstream signaling, proliferation (29, 31) and anti-viral responses (17, 18). Furthermore, IFNα subtypes have been shown to selectively activate different STAT and MAPK molecules, resulting in different anti-proliferative capacities against erythroleukaemia in an immune-deficient mouse model (20). We purposely selected individual IFNα subtypes based on previously reported characteristics that indicate potential differences in their biological activities (18). Interestingly, IFNα2, and IFNα9, which acted similarly in our study, are larger proteins and are located in a separate cluster on chromosome four to IFNα4, IFNα5, and IFNα6 (30, 31). It is possible that IFNα subtypes that cluster together may share similar biological activities. Evolutionary studies support this hypothesis demonstrating, at least in humans, that subtypes that cluster together are more closely related and therefore likely to share similar affinities for the IFNAR and activation of down-stream signaling pathways (32). A clearer understanding of the different IFNα subtype clusters may identify diverse biological roles for this family and provide ways to further enhance their potential to mediate effective anti-tumor immune responses.

Due to the potent ability of the IFNα family to mediate effective anti-tumor immune responses when expressed locally (5, 6, 33), we were interested in the effects of the different IFNα subtypes within the tumor microenvironment. While previous studies have demonstrated that overexpression of a single IFNα subtype by cancerous cells impairs tumor development (34–36), none of these studies have directly compared the immune-modulatory roles of individual IFNα subtypes head to head. Here, we provide evidence that distinct IFNα subtypes secreted locally in the tumor microenvironment vastly differ in their capacity to control tumor growth. IFNα4, IFNα5, and IFNα6 had the remarkable capacity to delay tumor growth for well-over 100 days. In contrast, IFNα2 and IFNα9 were able to completely abrogate tumor growth in a proportion of mice, a phenomenon that was dependent on an intact adaptive immune system. Remarkably, this protection could also be transferred to WT bystander tumor cells. Why IFNα2 and IFNα9 are able to control tumor growth compared to the other subtypes remains unclear. The loss of protection in RAG^o/o^ mice strongly suggests a critical role for T and/or B cells in mediating the anti-tumor protection observed. IFNα can act directly on CD8^+^ T cells to enhance cytotoxicity (37), increase pro-inflammatory cytokine production (38), promote persistence in the tumor microenvironment (34), and prolong survival of T cells (39). In support for a role of these subtypes in enhancing T cell immunity, adoptive transfer of T cells secreting IFNα9 significantly prolonged survival in mice bearing WT tumors over IFNα4-secreting T cells. Additionally, IFNα enhances NK cell activation (14), DC maturation (40), and B cell responses (41) to mediate effective immunity. Furthermore, a recent study demonstrated that IFNα2, IFNα4, IFNα6, and IFNα9 (but not IFNα1, IFNα5, or IFNα11) improved T cell cytotoxicity, demonstrating that subtype selection is an important consideration for optimal T cell effector function (19). Collectively, these findings provide a strong rationale for future studies aimed at elucidating the underlying mechanisms driving enhanced adaptive anti-tumor protection by IFNα2 and IFNα9.

Whilst we have observed a clear split between the different subtypes tested in this study, we cannot rule out that this effect may be driven by the amount of IFNα secreted locally in the tumor microenvironment. In the present study, we aimed to address dose by GFP reporter marker expression and a standard IFNα bioassay. Although our cell lines expressed similar levels of GFP (linked to IFNα expression), it is not possible to accurately determine the level of individual IFNα each cell line is producing using a reporter maker alone. The gold standard method to determine the biological activity of IFNα and to assess dose is the bioassay employed in this study, which is based on the ability of IFNα to inhibit viral infection. Indeed, differences in the anti-viral activity of supernatants derived from the various B16_IFNα cell lines were observed, despite comparable GFP expression. However, a major caveat of using this in our study is that the bioassay does not account for the varying anti-viral activities present amongst the IFNα family. Detection of protein by ELISA or other antibody-based assays is also commonly used to assess quantity, but are unsatisfactory as pan-IFNα antibodies cannot bind all members of the IFNα family. Thus, no commercially available reagents exist to confidently quantitate all the IFNα subtypes making it very difficult to accurately measure individual IFNα subtype protein. Assays designed to measure the quantity of all the discrete IFNα subtypes are certainly warranted, but our results point toward similar IFNα secretion among the B16_IFNα cell lines. It is also important to note that even if similar doses of IFNα are being produced in our model systems, it is unclear if the targets of IFNα, such as the immune compartment, respond equivalently, or if certain IFNα subtypes have a propensity for a particular immune subset as observed in viral responses (42). Nevertheless, we observe profound differences in anti-tumor control in our model systems. Clearly, under the right circumstances, IFNα is capable of significantly prolonging tumor growth, or completely abrogating development. Whether this is subtype-specific or dose-specific, these results highlight the complexity involved in analyzing the anti-tumor effects of IFNα and emphasize how critical it is to understand the mechanisms that underpin these differential anti-cancer responses.

Collectively, our data supports a crucial role for IFNα in the local tumor microenvironment for control of tumor growth. Clinically, IFNα is administered systemically and while this has resulted in moderate efficacy against melanoma (22), strategies to deliver IFNα directly to the tumor microenvironment are certainly warranted. Here, we show that engineering tumor-specific T cells to secrete IFNα9 prolonged survival over IFNα4, however this approach relies on constitutive IFNα secretion by the transferred T cells, most likely resulting in elevated levels of IFNα systemically. New advancements in cell engineering have made it possible to deliver compounds directly to the tumor microenvironment. These innovative approaches currently under development include the Notch AND-gate circuit system, in which T cells are engineered to express cytokines or antibodies only upon recognition of cognate antigen, thus confining their expression solely to the tumor microenvironment (43). Alternatively, the tumor-homing ability of TIE2^+^ monocytes (33) or the fusion of specific IFNα subtypes to tumor-specific antibodies (44) can be exploited, with both approaches resulting in potent anti-tumor responses.

In summary, our data provides evidence for diverse IFNα subtype-specific enhancement of the anti-cancer immune response. This work highlights the need to further research the role of the additional 11 IFNα subtypes in anti-cancer immune responses. In the clinic, only IFNα2 has been a gold standard for cancer treatment (22). Identifying which IFNα subtypes have increased immunomodulatory capacity and therapeutic efficacy compared to IFNα2 treatment in patients, will provide translational pathways for novel IFNα-based treatments.

## Data Availability Statement

The datasets generated for this study are available on request to the corresponding author.

## Ethics Statement

The animal study was reviewed and approved by Telethon Kids Institute Animal Ethics Committee (AEC#252, AEC#289, and AEC#325).

## Author Contributions

AB, VF, BF, and JW designed the experiments. AB, TW, KA, HN, LB, and SB performed experiments or analyzed data. AB, VF, BF, and JW were involved in data discussion and drafting the manuscript. AB and BF wrote the manuscript. AB, TW, VF, KA, HN, BW, SS, AM, BF, and JW edited the manuscript.

### Conflict of Interest

BW and SS are employed by the company PYC Therapeutics. The remaining authors declare that the research was conducted in the absence of any commercial or financial relationships that could be construed as a potential conflict of interest.

## Supplementary Material

Supplementary Figure 1 Generation of B16 melanoma cells engineered to secrete individual IFNα subtypes. **(A)** The gene sequences of IFNα2, IFNα4, IFNα5, IFNα6, and IFNα9 were amplified from pkCMV.int vectors by PCR. During amplification, EcoR1 restriction sites were added to the end of the IFNα gene sequences to allow ligation into the retroviral pMIG vector digested with EcoR1. **(B)** Characteristics of the individual murine IFNα subtypes. **(C)** Clustering and direction of transcription of the IFNα subtypes on murine chromosome 4.

Supplementary Figure 2 Proliferation of B16 cells engineered to secrete an individual IFNα subtype. **(A)** Engineered B16_GFP or B16_IFNα cells were mixed at a 1:1 ratio with **(B)** bystander B16_Cherry cells and were labeled with VPD450 proliferation dye before being seeded into a 6-well-plate. After 5 days, the cells were harvested and the expression of VPD450 was measured by flow cytometry. Each column represents mean ± SEM from three independent experiments and groups were compared using one-way ANOVA.

Supplementary Figure 3 Characterization of MHC-I expression on engineered and bystander B16 cells. Engineered B16_GFP and B16_IFNα cells were co-cultured with bystander B16_Cherry cells in the presence or absence of IFNγ for 48 h. The cells were harvested and the expression of H-2D^b^ and H-2K^b^ alleles of MHC-I were analyzed by flow cytometry. **(A)** Representative histograms of H-2D^b^ and H-2K^b^ expression on engineered cells in the presence and absence of IFNγ. Mean fluorescence intensities (MFI) of **(B)** H-2K^b^ and **(C)** H-2D^b^ expression on engineered B16 cells. **(D)** Representative histograms of H-2K^b^ and H-2D^b^ expression on bystander cells in the presence and absence of IFNγ. Mean fluorescence intensities (MFI) of **(E)** H-2K^b^ and **(F)** H-2D^b^ expression on bystander cells. Data was pooled from two independent experiments and compared using one-way ANOVA, ^*^*p* < 0.05 and ^***^*p* < 0.001.

Supplementary Figure 4 IFNα subtypes significantly delay tumor growth in WT mice. Tumor growth of individual WT mice inoculated subcutaneously with 5 × 10^5^ B16_GFP or B16_IFNα cells from four independent experiments (*n* = 10–18 per group).

Supplementary Figure 5 IFNα subtypes significantly delay tumor growth in RAG^o/o^ mice. Tumor growth of individual RAG^o/o^ mice inoculated subcutaneously with 5 × 10^5^ B16_GFP or B16_IFNα cells from two independent experiments (*n* = 9–12 per group).

Supplementary Figure 6 Delivery of IFNα9 into the tumor microenvironment by gB-specific CD8^+^ T cells impedes tumor development. **(A)** gBT.I cell activation and transduction began 2 days prior to subcutaneous tumor challenge of WT mice with 5 × 10^5^ B16_gB cells. Four days post-tumor inoculation, mice were subjected to 500 rads total body irradiation before receiving 3 × 10^6^ gBT.I cells (gBT.I-GFP) or gBT.I cells lacking the IFNAR (gBT.I_IFNAR^o/o^-GFP) not secreting IFNα, or secreting IFNα4, or IFNα9. **(B)** Tumor development was monitored over time and data pooled from two independent repeats (*n* = 5–10 mice per group). The IFNα9 cohort was compared to GFP alone and IFNα4 cohorts using the Log-Rank Mantel-Cox test, ^*^*p* < 0.05 for both comparisons.
