# Supplementary figures and images for: Diverse Anti-Tumor Immune Potential Driven by Individual IFNα Subtypes
Source: Front Immunol. 2020 Apr 3;11:542. doi: 10.3389/fimmu.2020.00542 (PMC7145903; doi:10.3389/fimmu.2020.00542)

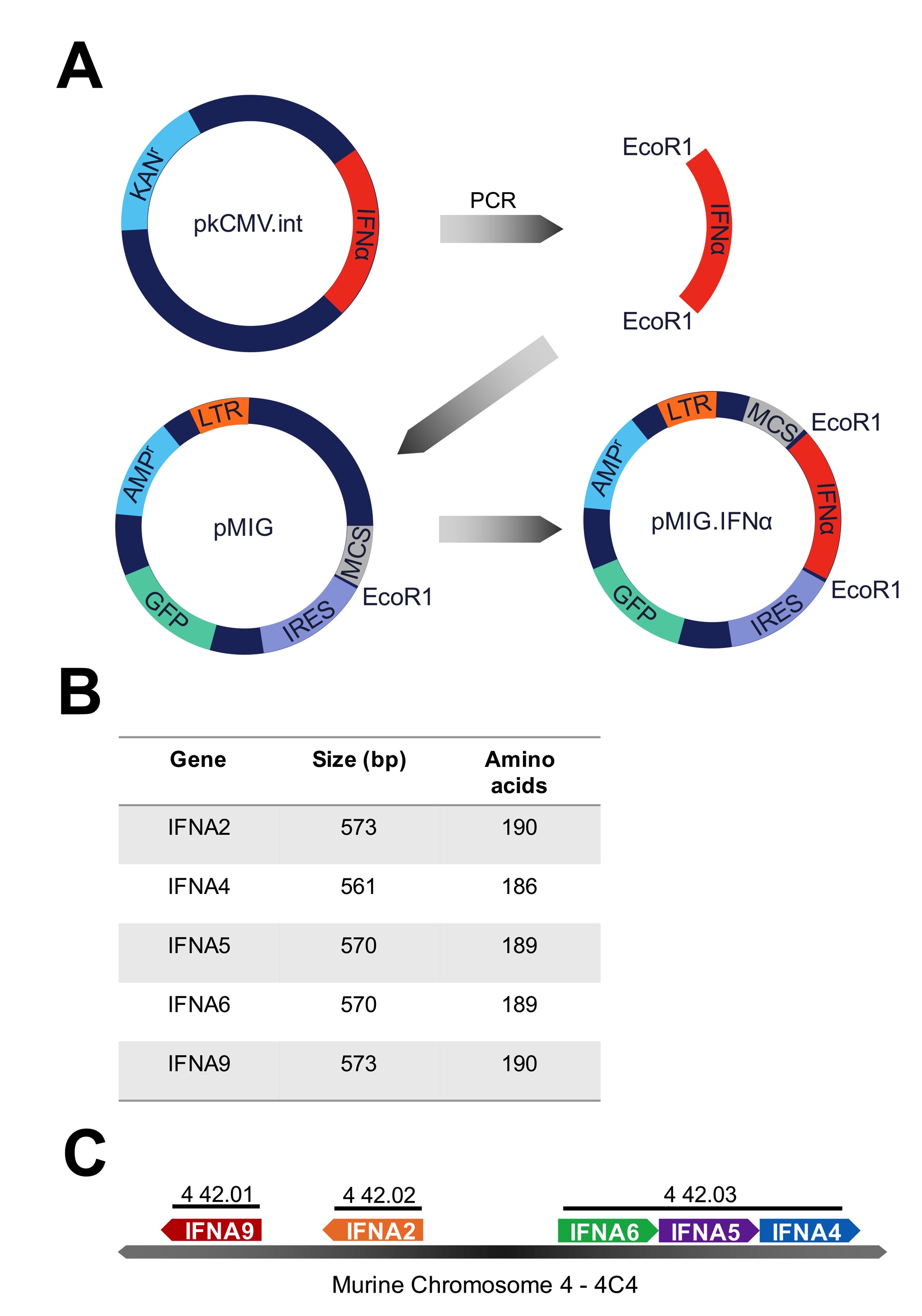

Supplement: Supplementary Figure 1 — Generation of B16 melanoma cells engineered to secrete individual IFNα subtypes. (A) The gene sequences of IFNα2, IFNα4, IFNα5, IFNα6, and IFNα9 were amplified from pkCMV.int vectors by PCR. During amplification, EcoR1 restriction sites were added to the end of the IFNα gene sequences to allow ligation into the retroviral pMIG vector digested with EcoR1. (B) Characteristics of the individual murine IFNα subtypes. (C) Clustering and direction of transcription of the IFNα subtypes on murine chromosome 4. [file Image_1.JPEG]

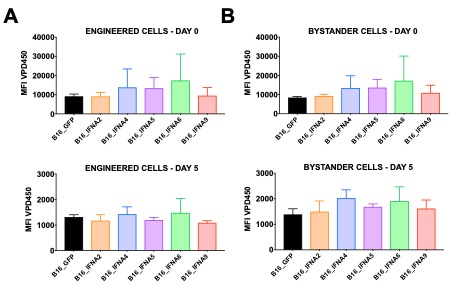

Supplement: Supplementary Figure 2 — Proliferation of B16 cells engineered to secrete an individual IFNα subtype. (A) Engineered B16_GFP or B16_IFNα cells were mixed at a 1:1 ratio with (B) bystander B16_Cherry cells and were labeled with VPD450 proliferation dye before being seeded into a 6-well-plate. After 5 days, the cells were harvested and the expression of VPD450 was measured by flow cytometry. Each column represents mean ± SEM from three independent experiments and groups were compared using one-way ANOVA. [file Image_2.jpeg]

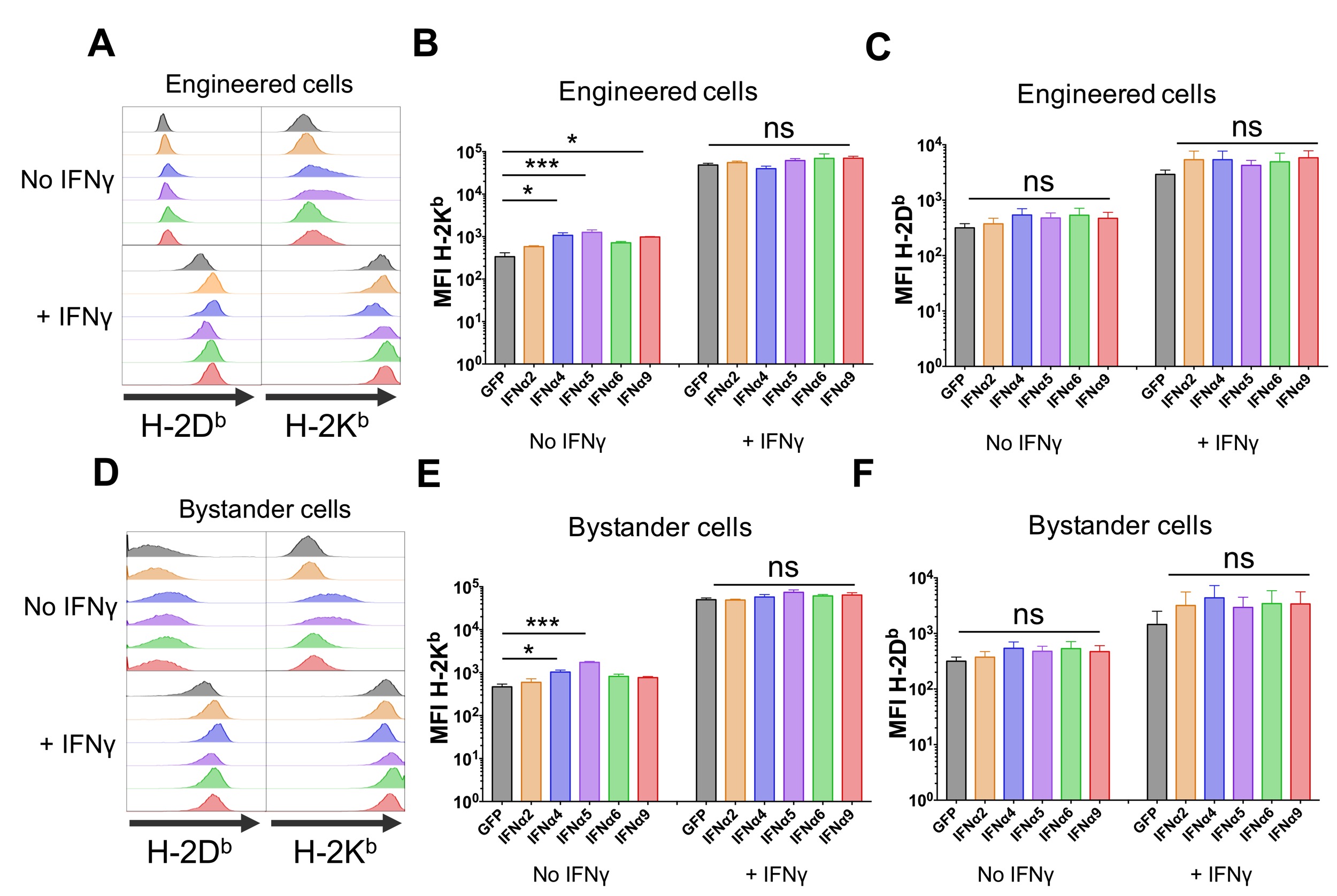

Supplement: Supplementary Figure 3 — Characterization of MHC-I expression on engineered and bystander B16 cells. Engineered B16_GFP and B16_IFNα cells were co-cultured with bystander B16_Cherry cells in the presence or absence of IFNγ for 48 h. The cells were harvested and the expression of H-2Db and H-2Kb alleles of MHC-I were analyzed by flow cytometry. (A) Representative histograms of H-2Db and H-2Kb expression on engineered cells in the presence and absence of IFNγ. Mean fluorescence intensities (MFI) of (B) H-2Kb and (C) H-2Db expression on engineered B16 cells. (D) Representative histograms of H-2Kb and H-2Db expression on bystander cells in the presence and absence of IFNγ. Mean fluorescence intensities (MFI) of (E) H-2Kb and (F) H-2Db expression on bystander cells. Data was pooled from two independent experiments and compared using one-way ANOVA, *p < 0.05 and ***p < 0.001. [file Image_3.JPEG]

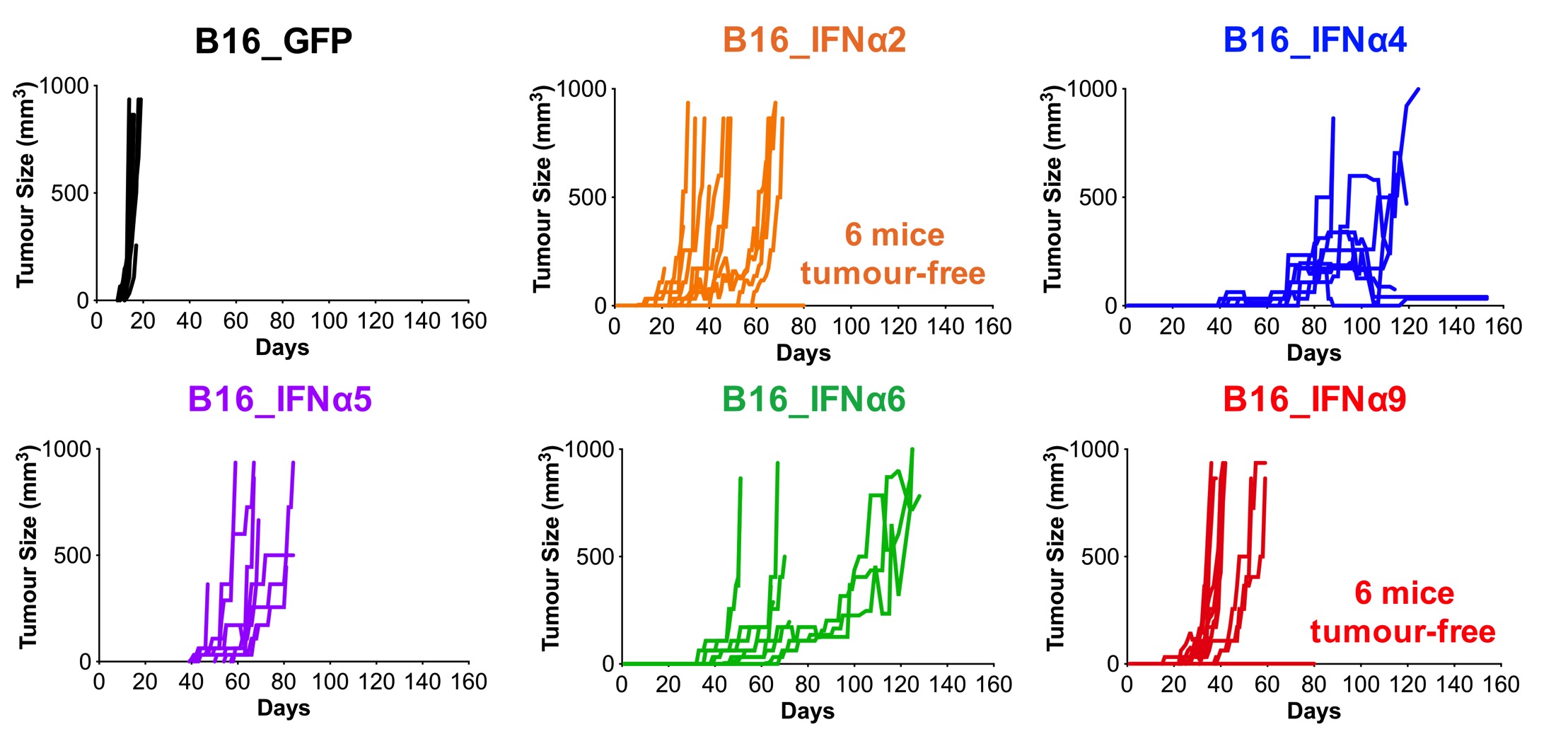

Supplement: Supplementary Figure 4 — IFNα subtypes significantly delay tumor growth in WT mice. Tumor growth of individual WT mice inoculated subcutaneously with 5 × 10^5^ B16_GFP or B16_IFNα cells from four independent experiments (n = 10–18 per group). [file Image_4.JPEG]

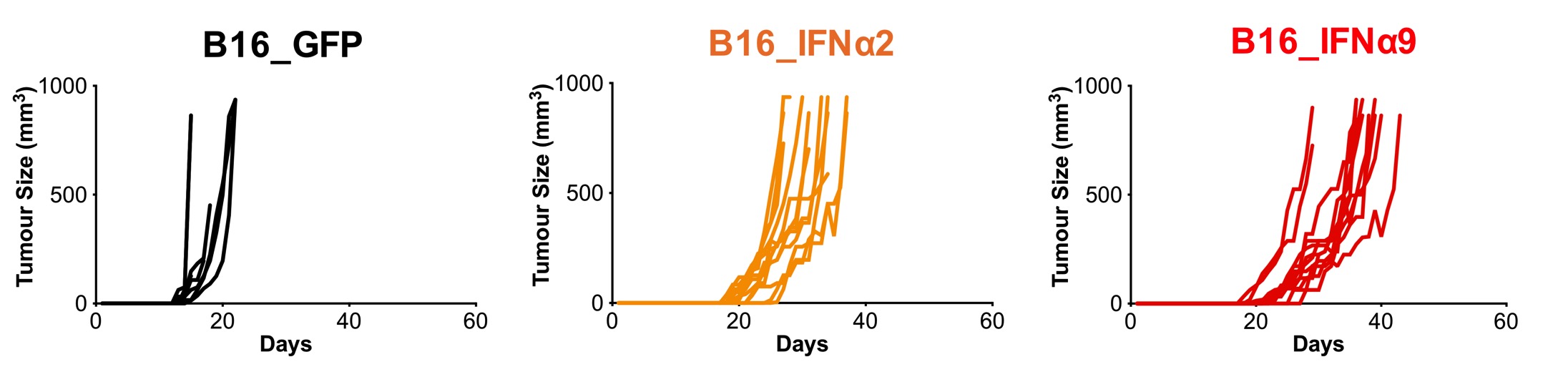

Supplement: Supplementary Figure 5 — IFNα subtypes significantly delay tumor growth in RAGo/o mice. Tumor growth of individual RAGo/o mice inoculated subcutaneously with 5 × 10^5^ B16_GFP or B16_IFNα cells from two independent experiments (n = 9–12 per group). [file Image_5.JPEG]

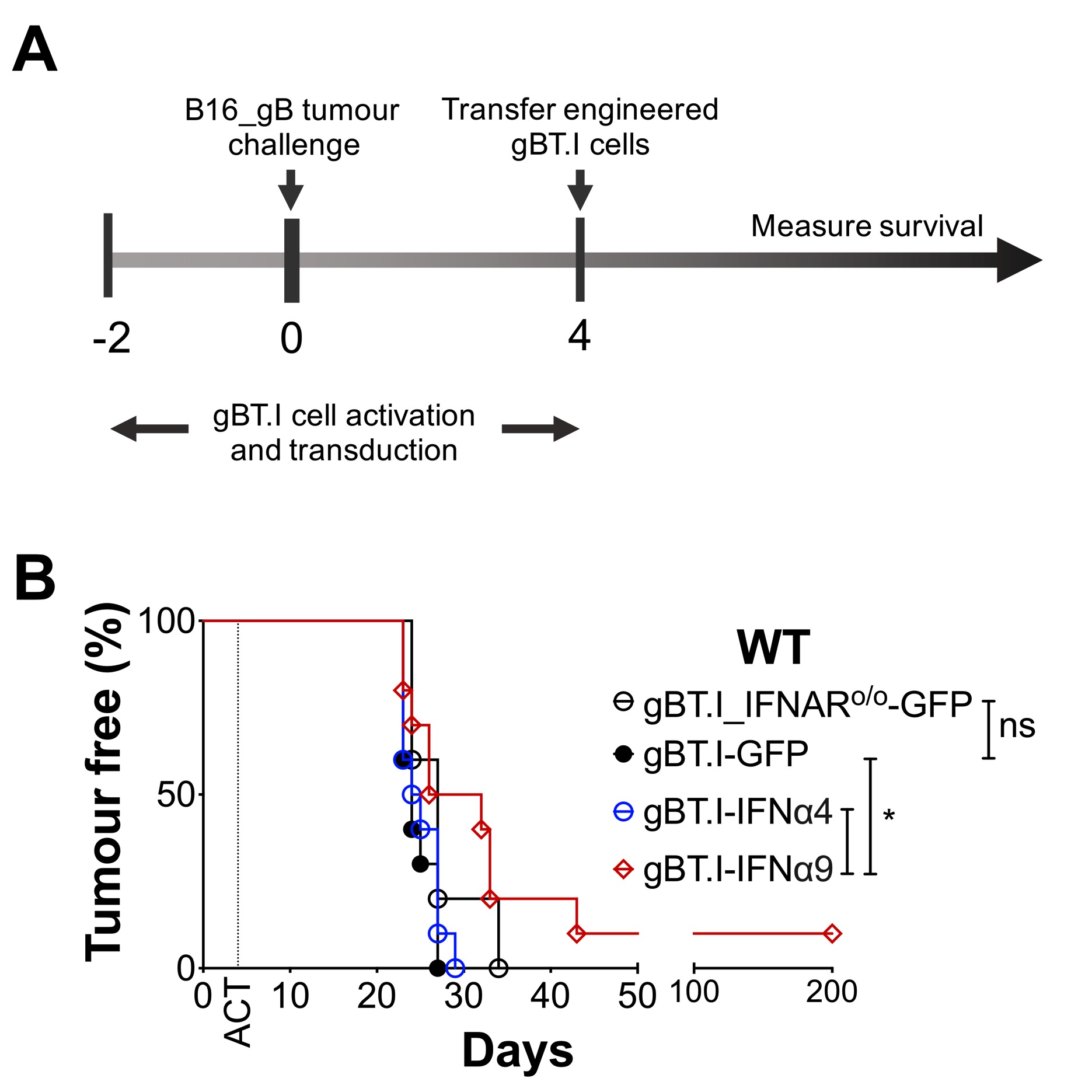

Supplement: Supplementary Figure 6 — Delivery of IFNα9 into the tumor microenvironment by gB-specific CD8+ T cells impedes tumor development. (A) gBT.I cell activation and transduction began 2 days prior to subcutaneous tumor challenge of WT mice with 5 × 10^5^ B16_gB cells. Four days post-tumor inoculation, mice were subjected to 500 rads total body irradiation before receiving 3 × 10^6^ gBT.I cells (gBT.I-GFP) or gBT.I cells lacking the IFNAR (gBT.I_IFNARo/o-GFP) not secreting IFNα, or secreting IFNα4, or IFNα9. (B) Tumor development was monitored over time and data pooled from two independent repeats (n = 5–10 mice per group). The IFNα9 cohort was compared to GFP alone and IFNα4 cohorts using the Log-Rank Mantel-Cox test, *p < 0.05 for both comparisons. [file Image_6.JPEG]
